# Effects of Dietary Milled Seed Mixture on Fatty Acid Status and Inflammatory Markers in Patients on Hemodialysis

**DOI:** 10.1155/2014/563576

**Published:** 2014-01-22

**Authors:** Danijela Ristic-Medic, Gordana Perunicic-Pekovic, Zorica Rasic-Milutinovic, Marija Takic, Tamara Popovic, Aleksandra Arsic, Marija Glibetic

**Affiliations:** ^1^Centre of Research Excellence in Nutrition and Metabolism, Institute for Medical Research, University of Belgrade, P.O. Box 102, 11000 Belgrade, Serbia; ^2^Department of Nephrology and Dialysis, Clinical Hospital Zemun, University of Belgrade, Belgrade, Serbia; ^3^Department of Endocrinology, Diabetes and Metabolism, Clinical Hospital Zemun, University of Belgrade, Belgrade, Serbia

## Abstract

*Background*. Plant seeds have gained interest for their health benefits due to their fatty acid content. The objective of this study was to determine the effects of dietary consumption of milled sesame/pumpkin/flax seed mixture on glycemic control, serum lipids, phospholipid fatty acid status, and inflammatory factors in patients on hemodialysis. *Methods*. Thirty patients with well nutrition status (18 male, 12 female) were enrolled in the study. Participants consumed 30 g of milled sesame/pumpkin/flax (6 g/6 g/18 g, resp.) seeds mixture added to their habitual diet. *Results*. Total n-6 and n-3 polyunsaturated fatty acids and levels of linoleic, dihomo-gamma-linolenic (DGLA), arachidonic, alpha-linolenic (ALA), eicosapentaenoic, docosapentaenoic, and docosahexaenoic (DHA) acid were increased after 12 weeks of supplementation. A significant decrease of the serum triglyceride level (*P* < 0.001), glucose, insulin, calculated IR HOMA (*P* < 0.05), and inflammatory markers (TNF-alpha, IL-6, and hs-CRP, *P* < 0.001) was observed after seed mixture treatment. The serum levels of CRP and TNF-alpha negative correlate with ALA, DHA, and DGLA. *Conclusion*. Results of this study indicated that dietary milled sesame/pumpkin/flax seed mixture added to a habitual diet lowered triglyceride and CRP, TNF-alpha, IL-6 levels, affect glycemic control and improved fatty acid profile and pruritus symptoms in hemodialysis patients.

## 1. Introduction

Fatty acid composition in serum phospholipids can be used not only as a biomarker of dietary fat intake quality, but also as an indicator of disease risk [1, 2]. In observational and intervention studies altered fatty acid composition of phospholipids linked with metabolic and cardiovascular diseases [2–4]. This suggestion implied a continued dietary supply. In our previous studies we showed that patients on hemodialysis have higher level of oleic (18 : 1 n-9) and lower levels of eicosapentaenoic (EPA) and docosahexaenoic (DHA) acids, with low levels of total n-6 and n-3 polyunsaturated fatty acid (PUFA) [5–8]. There are many limitations in the published studies which deal with changes in fatty acid (FA) metabolism and PUFA content in chronic renal failure. However, in general, it can be concluded that patients on hemodialysis need nutritional care programs with an adequate n-6/n-3 PUFA ratio in their diet [9, 10].

The precursor to long-chain n-3 fatty acid is alpha-linolenic acid (ALA), which is elongated to EPA and docosapentaenoic acid (DPA n-3), possibly more in women than in men [11, 12]. According to the available literature, further metabolism of DPA n-3 to DHA is very limited [12, 13]. However, the use of the plant-based n-3 fatty acid to be alternative to consummation of fish may be important for maintaining optimal EPA and DHA status in plasma and cell membranes. It is important to address that serum levels of ALA significantly increased after ingestion of flax oil and milled flaxseed whereas whole flaxseed, did not show the same effect [14, 15]. Flax, pumpkin, and sesame seeds have gained interest for their health benefits due to their fatty acid and lignan contents [16–18]. Flaxseed is also an excellent source of dietary fiber and contains protein and several micronutrients. Sesame and flax seeds are considerably different in the PUFA content, with a high ALA n-3 level (51–55%) in flaxseeds and high linoleic acid (LA) n-6 content in sesame seeds (44%). Since sesame seed is a rich source of n-6 FA and also monounsaturated FA (oleic acid 40%), in addition to diet, increased serum phospholipids LA levels as biomarker of intake are observed [19, 20]. The seeds of pumpkin are rich natural source of LA n-6 (40.4–55.6%), oleic acid, and antioxidant vitamins, such as carotenoids and tocopherol [21, 22]. The ideal ratio of LA n-6 and ALA n-3 in diet is not known, but ratio of 1 : 1-2 has been considered beneficial to health with effects on cell membrane fluidity and membrane function [23]. The balance required in the diet between n-6 and n-3 fatty acid is important due to their competitive nature and their different biological roles [24, 25].

Uremic pruritus is one of common symptoms in patients with end-stage renal disease (ESRD), with approximately 60–90% of patients on hemodialysis suffering from this problem [26, 27]. The pathogenesis of dry, scaly skin, and pruritus is not known but could be related to the abnormal fatty acid profile reported in these patients [10, 28]. It is shown that intervention with fish oil may improve symptoms of pruritus in hemodialysis patients [28].

In the latest prospective cohort study on inflammation and death risk, Noori et al. [29] concluded that a higher dietary n-6/n-3 PUFA ratio appears to be associated with inflammation and a trend toward higher risk of morality in hemodialysis patients. Inflammatory states in ESRD are associated with an elevation of C-reactive protein (CRP) and some proinflammatory cytokines such as interleukin-6 (IL-6) and tumor necrosis factor-alpha (TNF-alpha) [6, 8, 30]. IL-6 is reported to play a central role in the pathophysiology of the adverse effects of inflammation in ESRD patients. The increased activation of inflammatory cytokines such as IL-6 and TNF-alpha may cause muscle breakdown and hypoalbuminemia and may be involved in atherogenesis [31, 32].

The purpose of the present study was prospective evaluation of the effects of milled sesame/pumpkin/flax seed mixture dietary treatments on nutrition status, plasma lipids, phospholipids fatty acid profile, inflammatory markers, and symptoms of pruritus in patients on hemodialysis.

## 2. Patients and Methods

We cross-sectionally evaluated 65 patients on maintenance hemodialysis from the Department of Nephrology, University Hospital Zemun (Belgrade). For this study we included cohort group of 30 chronic renal failure patients (18 male, 12 female, mean age 55 ± 14 yr; range 42 to 64), who have been selected based on their well nutrition status. All patients had been on maintenance hemodialysis three times a week for at least 6 months and the mean duration of hemodialysis in included patients was 4.76 ± 3.38 yr. The duration of dialysis was defined as the number of months from the initiation of chronic hemodialysis to the time of laboratory data collection. The patients were clinically stable, with adequate nutrition status and with no recorded cardiovascular events (coronary heart disease and cerebrovascular disease). The mean body mass index (BMI) calculated from dry body weight was 22.63 ± 3.44 kg/m^2^ (range 19.8 to 27.3). Serum albumin concentration was 46.27 ± 7.22 g/L and serum CRP level was 6.26 ± 4.12 mg/mL. Patients were dialyzed with synthetic membranes and a bicarbonate dialysate with 1.25, 1.5, or 1.75 mmol/L calcium according to the serum calcium phosphate equilibrium and with obligatory use of 1.25-dihydroxy vitamin D3 to control the parathyroid hormone levels. The duration of hemodialysis was individually tailored (4–6 h weekly) to control body fluids and blood chemistries with the aim of achieving Kt/V > 1.2 (1.46 ± 0.13). Patients did not regularly receive epoetin therapy during that time. Antihypertensives were prescribed when it was necessary to obtain predialysis blood pressure < 160/90 mmHg. Patients with a history of nephrotic syndrome, diabetes mellitus, alcohol consummation, systemic illness, or any other disease that might influence lipid metabolism were excluded. None of the patients received lipid lowering drugs, L-carnitine, *β*-blockersor, vitamin B12 in the 3 months prior to entering the study. Patients received no other medications except multivitamins, calcitriol phosphate binders, and/or iron. Calcium carbonate was used to obtain predialysis serum phosphate level < 2.0 mmol/L. Patients maintained their habitual diets (35 kcal/kg bw, protein intake 1–1.2 g/kg bw, fats < 35% caloric intake) with sodium and potassium restriction. They had low habitual consumption of foods containing soy, fish intake once in two weeks, and no dietary supplementation of oil rich in long-chain fatty acid (fish, sesame, or linseed oil) as determined by diet assessment made at the time of recruitment. All patients were sedentary (<1 h/wk of physical activity), free of alcohol consumption, and nonsmokers. All study participants signed and provided an informed consent document. Study protocol was approved by the ethical review boards of the participating institutions in accordance with the principles of the Declaration of Helsinki.

### 2.1. Study Design

This study was a cross-sectional and follow-up dietary intervention study. Patients were instructed to mix 30 g per day of milled seed mixture containing 6 g sesame : 6 g pumpkin : 18 g flax (1 : 1 : 3) (company Beyond, Nis, Serbia; seed mixture was made in our laboratory) in 200 mL of fat-free milk (Imlek, Belgrade) in a single evening dose before dinner during the follow-up period of 12 weeks. In the present study, the test milled seed mixture was added to the habitual diet as they have already consumed at least 200 mL of fat-free milk a day at the moment they were recruited for our study. The participants were also instructed to continue to consume a usual amount of milk. Although no modification of the habitual diets for these patients was planned, they were instructed to keep their usual diets and level of physical activity relatively constant throughout the study and to maintain stable body weights. Fatty acid profile of ground seed mixture (analyzed with gas chromatography) was sum of palmitic and stearic fatty acid 18%, oleic acid (OA) 30%, LA 24%, and ALA 28%; polyunsaturated/saturated (PUFA/SFA) fatty acid ratio was 4 : 1 and n-6/n-3 ratio was 1 : 1. Daily fatty acid intake from milled seed mixture was palmitic acid 0.9 g, stearic 0.7 g, OA 3.9 g, LA 2.9 g, and ALA 3.0 g. Specific instructions were given not to take any new supplements or increase the amount of fish in the diet. The patients continued to consume (sunflower oil) n-6 PUFA cooking oils as part of their usual diet. The diet control was done as usual for all study participants by interview during hemodialysis.

For the purpose of this study, uremic pruritus was defined as pruritus appearing in close conjunction with the commencement of dialysis or appearing in conjunction with significant deterioration in renal function [33]. A questionnaire with issues to evaluate subjective feeling of presence and frequency of pruritus was given to patients at baseline and after intervention period. The pruritus questionnaire was used as the short form from Peck et al. [28] for assessment of uremic pruritus in part of the pruritus history. The questions covered the presence of dry skin or itching (yes/no), history of pruritus symptoms (1–4, from never to continuing daily, weekly, fortnightly, or monthly), severity (1–5, from mild to total restlessness), distribution (1–3, single location to generalized), and frequency of symptoms (number of episodes per 24 h).

### 2.2. Biochemical Determination

Blood samples were taken in the middle of week, after 12–14 h of overnight fasting and immediately prior to dialysis in the beginning of study and after 12 weeks. Serum samples were prepared by 4°C centrifugation of venous blood. Plasma triglyceride, glucose, and total cholesterol concentrations were measured by using standard enzyme color tests (EliTech Diagnostic. Sées, France); HDL cholesterol was determined by the enzyme procedure (EliTech Diagnostic. Sées, France) in supernatant after precipitation by the phosphotungstic acid and LDL cholesterol was estimated by using Friedewald formula. Total phospholipids were determined by the method of Zilversmit and Davis [34]. Insulin level was measured using the radioimmunoassay method (INEP Zemun, Belgrade, Serbia). Fasting insulin and glucose concentrations were used to calculate insulin resistance from the IR-HOMA model (insulin × glucose/22.5) [35].

Serum albumin concentration was determined by using bromcresol green reagent (EliTech Diagnostic. Sées, France). Serum iron was measured by a photometric color test for clinical chemistry analyzers (Olympus System Reagent; Olympus Diagnostica). IL-6 and TNF-alpha concentrations were measured in duplicate by Immunotech IL-6 immunoassays and Immunotech TNF-alpha immunoassays (Beckman Coulter, Fullerton, CA, USA), and hs-CRP was measured by the Olympus (LATEX) assay on the Olympus AU 400 analyzer (Olympus, PA, USA) by methods that have already been reported [7].

### 2.3. Fatty Acid Determination

Serum lipids were extracted according to method of Sperry and Brand [36] which uses chloroform-methanol mixture (2 : 1, v/v) with 10 mg/100 mL 2.6-di-tert-buthyl-4-methylphenol (BHT) added as an antioxidant. The phospholipids fraction was isolated from the lipid extract using one-dimensional thin-layer chromatography (TLC) in a neutral lipid solvent system hexane-diethyl ether-acetic acid (87 : 12 : 1, v/v/v) using Silica Gel GF plates (C. Merck, Darmstadt, Germany).

Methyl esters of phospholipids FA were prepared by methods that have already been reported [37]. Fatty acid methyl esters derivatives were analyzed by gas chromatography using Varian GC (model 3400, Varian Associates) equipped with SP-2330 (30 m × 0.53 mm i.d., film thickness 0.5 *μ*m; Supelco Inc., Bellefonte, PA) fused silica capillary column. Analysis was performed in duplicate for each sample. Individual FA methyl esters were identified by comparing peak retention times with authentic standards (Sigma Aldrich, Germany) and/or the PUFA-2 standard mixtures (Supelco Inc., Bellefonte, PA). The results were expressed as the relative percentage of total identified FA.

### 2.4. Statistical Analysis

All the results are expressed as the mean ± SD. Before being statistically analyzed, all data were examined for normality by using the Shapiro-Wilks test. Differences in the absolute changes in fasting plasma triglycerides, total cholesterol, HDL cholesterol, LDL cholesterol, albumin, creatinine, hemoglobin, hs-CRP, TNF-alpha, IL-6, glucose, insulin, iron, calculated IR HOMA, and FA profile of serum phospholipids over the 12-week period of milled seed mixture supplementation were determined using independent-sample *t*-tests or Mann-Whitney *U* test for variables with skewed distribution. The Pearson correlation coefficients were computed for examination of the relation between inflammatory factors and fatty acid composition of serum phospholipids. The differences were considered significant at *P* ≤ 0.05.

## 3. Results

### 3.1. Subject Characteristics

The baseline characteristics of the study participants are presented in Table 1. All participants completed the study and there were no withdrawals. There were no significant differences in the anthropometrical characteristics of hemodialysis patients before and after 12 weeks of milled seed mixture dietary treatments. Most patients (20 participants) had suffered from pruritus for periods ranging from 6 months to 1 year. In most patients (60%), pruritus symptoms were aggravated during the night sleeping period compared to rate of pruritus symptoms during the daytime. Seed mixture added to diet improves the symptoms of pruritus in all patients. The scores for severity, distribution, and frequency of pruritus symptoms on the questionnaire showed a great improvement. Systolic and diastolic blood pressure were significantly decreased after 12 of weeks of treatment (*P* < 0.01). We also found significant decrease of creatinine level (*P* < 0.05) at the end of the study. This seed mixture was well tolerated with no significant side effect in hemodialysis patients.

### 3.2. Serum Lipids

Effect of milled seed mixture dietary treatments on mean serum lipid values for all participating patients and percentage change are shown in Table 2. After 12 weeks of consuming milled sesame/pumpkin/flax seed mixture in the hemodialysis patients, total and LDL cholesterol concentrations had decreased by 7% and 5% (*P* < 0.05, *P* > 0.05), respectively (Table 2). The Tg/HDL (*P* < 0.01) ratio significantly decreased but TC/HDL and LDL/HDL cholesterol ratios decreases remained non statistic. Serum HDL cholesterol did not differ from baseline values throughout either feeding period. During 12 weeks of seed mixture consumption, serum plasma triglyceride levels decreased by 30% (*P* < 0.001) compared with baseline values (week 0) (Table 2).

### 3.3. Fatty Acid Profile of Serum Phospholipids

The fatty acid profiles of serum phospholipids before and after treatment are shown in Table 3. Saturated 16 : 0 (*P* < 0.01), 18 : 0 (*P* < 0.01), and total SFA (*P* < 0.001) were significantly decreased and oleic acid (*P* < 0.05) and total MUFA increased (*P* < 0.01) in serum phospholipids after treatments. Percentages of LA (*P* < 0.01), dihomo-*γ*-linoleic (DGLA; 20 : 3n-6) (*P* < 0.01), AA (*P* < 0.05), ALA (*P* < 0.05), EPA (*P* < 0.05), DPA n-3 (*P* < 0.05), and DHA (*P* < 0.01) in serum phospholipids were significantly higher after 12 weeks of intervention. Consistent with the previous were the increases in n-6 PUFA n-3 PUFA, total PUFA, and PUFA/SFA ratios as well (*P* < 0.001 for all mentioned parameters) (Table 3).

### 3.4. Glucose Tolerance and Inflammatory Markers

A significant decrease of the glucose, insulin, calculated IR HOMA (*P* < 0.05 for all mentioned variables), and inflammatory markers (TNF-alpha, IL-6, hs-CRP, *P* < 0.001) was observed after dietary intervention (Table 1). The serum levels of CRP negative correlate with ALA, DHA, DGLA, and AA in serum phospholipids (Table 4). There was very strong negative correlation between serum TNF-*α* and ALA, DHA, LA, and DGLA. The serum levels IL-6 negative correlate with ALA, DPAn-3, DGLA, and AA in serum phospholipids (Table 4).

## 4. Discussion

The results clearly demonstrate that dietary treatment with milled sesame/pumpkin/flax seeds mixture (30 g; rich in n-6 and n-3 FA) for 12 weeks has the capacity to improve traditional lipid levels, inflammatory markers, and serum fatty acid composition as biomarker of status in hemodialysis patients. We also showed the efficacy of increasing levels of not only EPA, but also DHA in plasma phospholipids by providing its precursor ALA (3.0 g) from seed mixture. That was inconsistent with reports that EPA can limitedly be converted to DHA [12, 38]. Therefore, it is important to emphasize that our patients have low intake of fish, once every two weeks, as poor eating patterns of people in our region [25]. Human clinical studies show that an increase in dietary ALA leads to significant increases in ALA, EPA, and DPAn-3 in the blood [39, 40] and they are carried out using daily intakes of > 5 g ALA. It is well known that fatty acid composition of the serum phospholipids clearly reflects the change of dietary habits [41, 42]. This is proved by analysis of fatty acid status in our hemodialysis patients after dietary treatment. Our findings for changes of LA levels in serum phospholipids, LA being the most represented fatty acid (2.9 g) in our seed mixture beside ALA (3.0 g), are consistent with Okita et al. [42] who reported that the LA levels in serum phospholipids appeared to reflect the dietary LA content.

As mentioned above, dietary LA and ALA serve as the precursors for the n-6 and n-3 series of PUFA, respectively, and play an important role in modulation of cardiovascular, inflammatory diseases, immune system, and blood pressure regulation [4, 24, 43]. Their products modulate biosynthesis of potent cellular mediators, eicosanoids. Therefore, increased consumption of food with high n-6/n-3 PUFA ratio leads to thrombosis and inflammatory reactions, while high n-3/n-6 ratio has cardioprotective, antithrombotic, and anti-inflammatory action [4, 44]. However, no reports are available on the effects of plant flax/pumpkin/sesame seed mixture (rich in n-6 and n-3 FA), used as dietary supplement on cardiometabolic risk factors in patients with renal disease compared to many studies with marine n-3 fatty acid. Adding seed mixture to the diets of hemodialysis patients resulted in large drops in blood pressure (BP) of around 11 mmHg systolic and 6 mmHg diastolic after 12 weaks. The evidence for a hypotensive effect of flax or its components is unconvincing [45, 46]. However, dietary supplementation with 8 g/day ALA for 12 weeks used by Paschos et al. [47] lowers both systolic and diastolic BP in dyslipidemic men with magnitude of the hypotensive effect (5 mmHg or 3–6%). Study conducted by Gossell-Williams et al. [48] showed that, in women, receiving pumpkin seed oil (2 g/day) decreased diastolic BP. The antihypertensive effect of seed mixture (rich in n-6 and n-3 FA) may be caused by changes in prostaglandin synthesis, by alteration of membrane fatty acid composition and by subsequent changes in membrane functions [46]. Some investigators have shown that flaxseed supplementation (30 g/day) lowers serum creatinine in healthy subject (32 g/day) [45] and has renoprotective effects in patients with lupus nephritis [49]. In the present study, adding milled sesame/pumpkin/flax seeds mixture (30 g; rich in n-6 and n-3 FA) in the habitual diet is accompanied with reduction of serum creatinine levels. It is well known that there was a strong association of severe pruritus with depression symptoms, poorer sleep, and dry skin in hemodialysis patients [27]. The decrease in frequency symptoms of pruritus in our patients indicated that balanced dietary n-6 and n-3 fatty acid intake improves symptoms of pruritus.

This is the first exploration of the influence of added milled sesame/pumpkin/flax seeds mixture in diet on serum lipids and fatty acid status, so we have a coupled effect of these plant food components. Flaxseed oil (2.4–3.6 g/day) has not shown any hypolipidemic effect in supplementation trials [50]. However, considering the publications, ground flaxseed (30–50 g/day) [16, 17, 51] and sesame seed (50 g/day) [19] have been shown to reduce blood lipids levels. In this regard, meta-analyses on eleven studies examining the effect of whole flaxseed supplementation on lipids levels reported that interventions reduced total and LDL cholesterol by 0.21 mmol/L and 0.16 mmol/L, respectively [52]. It is noteworthy, that the high content of dietary fiber and LA in the sesame seed underlies its capacity to lower serum cholesterol levels [18].

In animal models of diabetes, pumpkin seeds supplementation decreased serum glucose, triglyceride and cholesterol, and CRP levels [53]. It is assumed that the possible potential mechanism of lipid lowering by pumpkin is probably due to fibers effects. It is well known that dietary fiber reduces plasma LDL cholesterol levels, by inhibiting the absorption of bile acids and cholesterol, and increases the activity of LDL receptors. Recently, Makni et al. [54] have reported that flax and pumpkin seed mixture supplemented in the diet of diabetic rats has hypoglycemic, hypolipidemic, and nephroprotector effect. In the present study we noticed significant reduction in cholesterol by 8% in hemodialyses patients, after dietary treatment with milled sesame/pumpkin/flax seed mixture. LDL cholesterol is the primary target in cholesterol lowering efforts in patients. In general, 1% increase in the LDL cholesterol level may lead to 2-3% increase in coronary artery disease risk [55]. Results of our study showed that added milled sesame/pumpkin/flax seed mixture in diet decreases slightly but not significantly serum LDL cholesterol by 5%. Meta-analysis [56] based on population prospective studies showed that 1 mmol/L increase in serum triglyceride was associated with a 32% increase in a risk factor for cardiovascular disease (CVD). In the present study, we have 30% (0.6 mmol/L) decreases in serum triglyceride level in hemodialysis patients after dietary treatment with milled sesame/pumpkin/flax seed mixture. However, the data related to the protective role of ALA (3.0 g content of ALA in our flax seed mixture) are less definitive than those of long chain n-3, EPA, and DHA [57, 58]. This reduction in fasting serum triglyceride during our intervention was in contrast with the conclusion that the plant-derived n-3 fatty acid is not equivalent to marine-based fatty acid in terms of lowering triglyceride levels [52]. Our study results indicate that sesame/pumpkin/flax diet treatment in hemodialyzed patients could be beneficial in prevention of CVD as triglyceride level is an important factor that has been implicated in the pathogenesis of cardiovascular complication.

It was suggested that plant-based n-3 fatty acids might influence insulin secretion in vivo and improve glucose use and efficiency [59]. Findings from limited studies suggest that flax meal improves insulin sensitivity, likely because of its soluble fiber content, which may delay postprandial glucose absorption in the gut. However, the trial by McManus et al. [60] revealed no effect of n-3 fatty acids from flaxseed oil on HbA1c, fasting glucose, or insulin levels in patients with well-controlled type 2 diabetes. Dietary milled sesame/pumpkin/flax seed mixture intakes in our study affect glycemic control, significant decreased glucose levels, insulin, and calculated IR HOMA in hemodialysis patients.

CRP plays an important role in atherogenesis [61]. Increased CRP levels exhibit synergy with concurrent hypercholesterolemia to increase CVD risk in both men and women [61]. Several studies have shown that CRP is inversely associated with EPA and DHA levels in both healthy subjects and in patients with stable coronary artery disease [62, 63]. Our results demonstrate that a dietary treatment with milled sesame/pumpkin/flax seeds decreased CRP, IL-6, TNF-alpha levels, and the changes in serum ALA and DHA levels, and ALA+ DPA + EPA + DHA (total n-3 FA) were inversely associated with changes in CRP and TNF-alpha. Therefore, decreasing CRP levels, by dietary interventions, can improve the lipid responses, thereby reducing overall CVD risk. In diseases in which inflammation leads to adverse effects on the progression of clinical parameters, such as renal failure, it is necessary fact that dietary intervention that suppresses the production of proinflammatory markers should be included as part of the prevention. Nonetheless, in a recent systematic review, a possible benefit of dietary ALA consumption in reducing sudden death and nonfatal myocardial infarction was deduced [44]. In line with this and because it has been shown that the levels of serum fatty acids vary according to the choice of different foods in the diet, dietary milled sesame/pumpkin/flax seed mixture supplemented to the habitual diet has beneficial effects on reducing CVD risk in hemodialysis patients. For the interpretation of the results, there should be mentioned that there was no placebo group as a control and that dietary fatty acid intake was not assessed by commonly used dietary assessment methods. Instead, compliance was measured as a change in the serum phospholipid fatty acid composition. Yet, despite these limitations, the status biomarker of dietary fatty acid intake showed the good patients' compliance, which means that the participants adhered to the dietary intervention treatment. In conclusion, milled 6 g sesame/6 g pumpkin/18 g flax seeds mixture per day have possible health-promoting effects due to increased levels of n-3 fatty acids in the serum and decreased inflammatory markers. Further well-designed and controlled studies are needed to determine if using seeds mixture might lead to prevention or regression of atherosclerosis and the resolution of some clinical problems in hemodialyzed patients.

## Figures and Tables

**Table 1:** The baseline characteristics of study participants and responsiveness to milled seed mixture after twelve weeks.

Variable	Before treatment	After treatment
Age	55 ± 14	
Gender (M/F)	30 (18/12)	
Duration of HD (years)	4.76 ± 3.38	
BMI (kg/m^2^)	22.63 ± 3.44	22.81 ± 3.62
SBP (mmHg)	155 ± 14	144 ± 13**
DBP (mmHg)	92 ± 9	86 ± 10**
WBC 10^9^/L	6.40 ± 1.46	5.74 ± 1.46
Hematocrit L/L	0.27 ± 0.04	0.26 ± 0.06
Serum creatinine (µmol/L)	785 ± 158	722 ± 156*
Serum urea (µmol/L)	20.56 ± 2.30	19.41 ± 2.34
Serum iron (µmol/L)	13.67 ± 4.77	14.49 ± 4.34*
Serum albumin (g/L)	46.27 ± 7.22	43.74 ± 4.05
Serum glucose (mmol/L)	4.76 ± 1.18	4.28 ± 0.82*
Serum insulin (pg/mL)	23.98 ± 9.42	20.28 ± 6.36*
IR HOMA	5.09 ± 2.82	3.83 ± 1.35*
TNF (mU/L)	1.86 ± 0.90	0.72 ± 0.59***
IL-6 (pg/mL)	2.63 ± 1.45	0.55 ± 0.34***
hs-CRP (mg/L)	6.26 ± 4.12	3.06 ± 1.34***

The values are means ± SD. *P ≤ 0.05, **P ≤ 0.01, and ***P ≤ 0.001 significantly different from baseline.

**Table 2:** Baseline serum lipids profile responsiveness to milled seed mixture after twelve weeks.

Serum lipids	Baseline (mmol/L)	After treatment (mmol/L)	Change %	Probability
Triglycerides	2.06 ± 1.20	1.44 ± 0.92	−30	*P* ≤ 0.01
Total cholesterol	5.47 ± 1.35	5.01 ± 0.81	−7	*P* ≤ 0.05
HDL cholesterol	1.30 ± 0.11	1.27 ± 0.14	−2	ns
LDL cholesterol	3.24 ± 1.08	3.08 ± 0.40	−5	ns
TC/HDL ratio	4.26 ± 1.27	4.01 ± 0.75	−6	ns
LDL/HDL ratio	2.53 ± 0.98	2.47 ± 0.47	−2	ns
Tg/HDL ratio	1.61 ± 0.98	1.17 ± 0.76	−27	*P* ≤ 0.01
Total phospholipids	2.87 ± 0.74	2.50 ± 0.51	−13	ns

The values are means ± SD.

**Table 3:** Serum phospholipids fatty acid composition at baseline and changes induced by dietary intervention.

Serum fatty acids	Baseline	After treatment
16:0	25.32 ± 2.39	23.87 ± 2.09**
18:0	16.44 ± 1.74	15.05 ± 1.02**
**SFA**	41.76 ± 3.22	38.93 ± 2.34***

16:1n-7	0.39 ± 0.16	0.36 ± 0.15
18:1n-9	13.83 ± 1.68	14.92 ± 0.86*
**MUFA**	14.59 ± 1.66	15.28 ± 0.94**

18:2n-6 LA	24.49 ± 2.62	25.73 ± 1.85**
20:3n-6	2.57 ± 0.79	2.96 ± 0.70**
20:4n-6	11.63 ± 2.31	12.77 ± 2.56*
22:4n-6	0.56 ± 0.14	0.61 ± 0.11
**n-6**	39.25 ± 3.25	42.06 ± 2.72***

18:3n-3 LNA	0.08 ± 0.03	0.12 ± 0.02*
20:5n-3 EPA	0.18 ± 0.05	0.22 ± 0.08*
22:5n-3	0.46 ± 0.07	0.51 ± 0.10*
22:6n-3 DHA	2.75 ± 0.33	2.99 ± 0.39**
**n-3**	3.46 ± 0.37	3.84 ± 0.42***

**PUFA**	42.71 ± 3.39	45.90 ± 2.69***
**n-6/n-3 ratio**	11.34 ± 1.25	10.91 ± 1.59
**PUFA/SFA ratio**	1.03 ± 0.15	1.10 ± 0.14***

The values are means ± SD. *P ≤ 0.05, **P ≤ 0.01, and ^∗ ∗ ∗^ *P* ≤ 0.001 significantly different from baseline.

**Table 4:** Correlation matrix between inflammatory markers and serum phospholipids fatty acid percentage in dietary intervention treatments.

	CRP	IL-6	TNF	ALA	EPA	DPA	DHA	n-3	LA	DGLA	AA	n-6
CRP	—	0.455**	0.392**	−0.369**	−0.112	−0.224	−0.282*	−0.343**	−0.123	−0.523**	−0.255***	−0.379**
IL-6		—	0.780***	−0.269*	−0.185	−0.286*	−0.213	−0.308*	−0.232	−0.262*	−0.293*	−0.438**
TNF			—	−0.587***	−0.140	−0.197	−0.376**	−0.403**	−0.605***	−0.319**	−0.203	−0.370**

*P < 0.05, **P < 0.01 and ***P < 0.001.
